# The genome sequence of the Tansy Beetle, *Chrysolina graminis *(Linnaeus, 1758)

**DOI:** 10.12688/wellcomeopenres.24287.1

**Published:** 2025-06-02

**Authors:** Geoff S. Oxford, Natural History Museum Genome Acquisition Lab, Darwin Tree of Life Barcoding collective, Wellcome Sanger Institute Tree of Life Management, Samples and Laboratory team, Wellcome Sanger Institute Scientific Operations: Sequencing Operations, Wellcome Sanger Institute Tree of Life Core Informatics team, Tree of Life Core Informatics collective, Darwin Tree of Life Consortium

**Affiliations:** 1 Department of Biology, University of York, York, England, UK

**Keywords:** Chrysolina graminis, Tansy Beetle, genome sequence, chromosomal, Coleoptera

## Abstract

We present a genome assembly from a female specimen of *Chrysolina graminis* (Tansy Beetle; Arthropoda; Insecta; Coleoptera; Chrysomelidae). The assembly contains two haplotypes with total lengths of 1,467.20 megabases and 1,455.42 megabases. Most of haplotype 1 (98.03%) is scaffolded into 12 chromosomal pseudomolecules, including the X sex chromosome. Haplotype 2 was assembled to scaffold level. The mitochondrial genome has also been assembled, with a length of 17.07 kilobases.

## Species taxonomy

Eukaryota; Opisthokonta; Metazoa; Eumetazoa; Bilateria; Protostomia; Ecdysozoa; Panarthropoda; Arthropoda; Mandibulata; Pancrustacea; Hexapoda; Insecta; Dicondylia; Pterygota; Neoptera; Endopterygota; Coleoptera; Polyphaga; Cucujiformia; Chrysomeloidea; Chrysomelidae; Chrysomelinae; Chrysomelini; *Chrysolina*; *Chrysolina graminis* (Linnaeus, 1758) (NCBI:txid111401)

## Background

*Chrysolina graminis*, the Tansy Beetle, is a relatively large (10 mm) leaf-beetle with most of its visible surfaces appearing iridescent green or bronze, which shimmer in sunlight. Globally. it is widely distributed throughout the Palaearctic, with records from most of West, Central, Northern and Eastern Europe and, in Asia, east to Mongolia and north to Siberia. It seems to be declining across most of its range except, perhaps, in the Netherlands.

**Figure 1.**
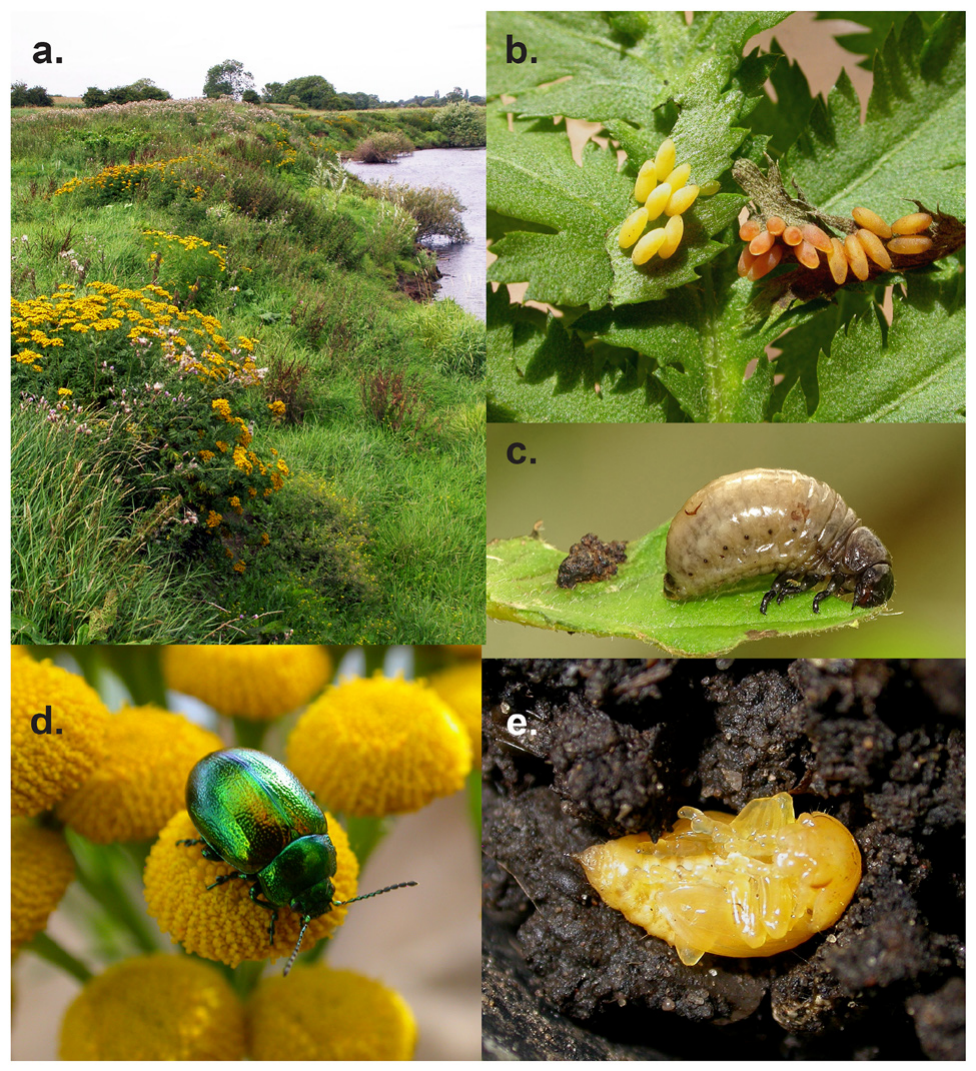
Images of the Tansy beetle and its environs in York (Photographs by Geoff Oxford). Clockwise from top left: Clumps of Tansy along the Yorkshire Ouse, Tansy Beetle eggs, showing colour variation; Tansy Beetle larva – (Photo by Julian Hodgson); Tansy Beetle pupa - Geoff Oxford; Tansy Beetle adult female on its York foodplant - Geoff Oxford. Images of the Tansy beetle ( *Chrysolina graminis*) (Photographs by Geoff Oxford and Julian Hodgson). **a**) Clumps of Tansy ( *Tanacetum vulgare*), **b**) Tansy beetle eggs showing colour variation., **c**) Tansy beetle larva (photograph by Julian Hodgson). **d**) Adult female Tansy beetle on Tansy flowers. **e**) Tansy beetle pupa.

Its life cycle has been well-established for the York population ( Chapman *et al.*, 2006; Oxford *et al.*, 2003). From early April onwards, overwintered females mate and begin to lay creamy yellow eggs in small batches (median clump size is 5 to 6 eggs; Oxford & Oxford, 2022). These are usually placed on the food plant, but sometimes on none-food plants nearby ( Figure 1a and b). The eggs hatch into beige, slug-like larvae ( Figure 1c) that progress through four instars until, towards the end of June, they pupate in soil at the base of the plants ( Figure 1e). By the end of June, most overwintering adults will have died. Adult beetles emerge a month later and feed ( Figure 1d); some mating also takes place at this time of year. Towards the end of September, the adults return underground to overwinter. Beetles in August and September have functioning wings and wing muscles and on hot days are capable of short, but seemingly reluctant, flights. Over winter, however, the wing muscles atrophy ( Oxford *et al.*, 2021); most movement between food plants is by walking ( Chapman *et al.*, 2007).

Up until 2014, the population of *C. graminis* living along the banks of the Yorkshire River Ouse was thought to be the only one extant in the British Isles. In his Species Status Review of leaf beetles in Britain, Hubble (2014) was intending an IUCN category of Critically Endangered (CR). Just before publication, however, a population was rediscovered at Woodwalton Fen, a NNR in the East Anglian Fens, which immediately precluded this status. Currently the species is considered Endangered (EN). In 2018, a third population was found at Welney on the Ouse Washes in West Norfolk. Despite its rarity classification, the beetle has no formal protection in law.

Since 2008, the Tansy Beetle Action Group (TBAG) has been coordinating research on *C. graminis*. As part of this programme, since 2010, annual population surveys have been made along some 45 km (90 km of bank) of the Yorkshire Ouse, which encompasses the beetle’s entire range on the river ( Oxford, 2021). In 2023, the population was estimated at some 90,000 beetles, although numbers in other years have been variable and often much lower. TBAG has produced two Conservation Action Plans for the York population, the second of which spans 2023–2027 and is available at https://tinyurl.com/33dvfxya. Around York, a small number of ark populations has been established beyond the River Ouse floodplain as insurance against catastrophic summer flooding.

Research suggests that the York and East Anglian Fenland populations may differ in aspects of their biology. Their food plants are different, with the York population living largely on Tansy *Tanacetum vulgare* while in the Fens several, typical wetland, species are eaten (which do not include Tansy). York beetles fail to thrive when reared on single-species Fenland plants ( Oxford & Oxford, 2022). There are hints that the phenology of at least the York and Woodwalton Fen populations are slightly shifted, and there may be differences in where beetles pupate and overwinter because of the different hydrological conditions they encounter ( Oxford, 2021). The two Fenland populations seem to be considerably smaller than that on the Yorkshire Ouse. They too are monitored and are the subject of current research.

The genome of *Chrysolina graminis* was sequenced as part of the Darwin Tree of Life Project, a collaborative effort to sequence all named eukaryotic species in the Atlantic Archipelago of Britain and Ireland. The *C. graminis* genome, from a specimen collected in York, will provide a vital benchmark against which to compare the genomes of specimens from the other two known British populations, and further afield in Europe. This knowledge will be crucial in determining whether the York and Fenland populations should be considered as evolutionary distinct groups, as suggested by currently known differences between them (see above), and inform future conservation actions, such as translocations.

## Genome sequence report

### Sequencing data

**Figure 2.**
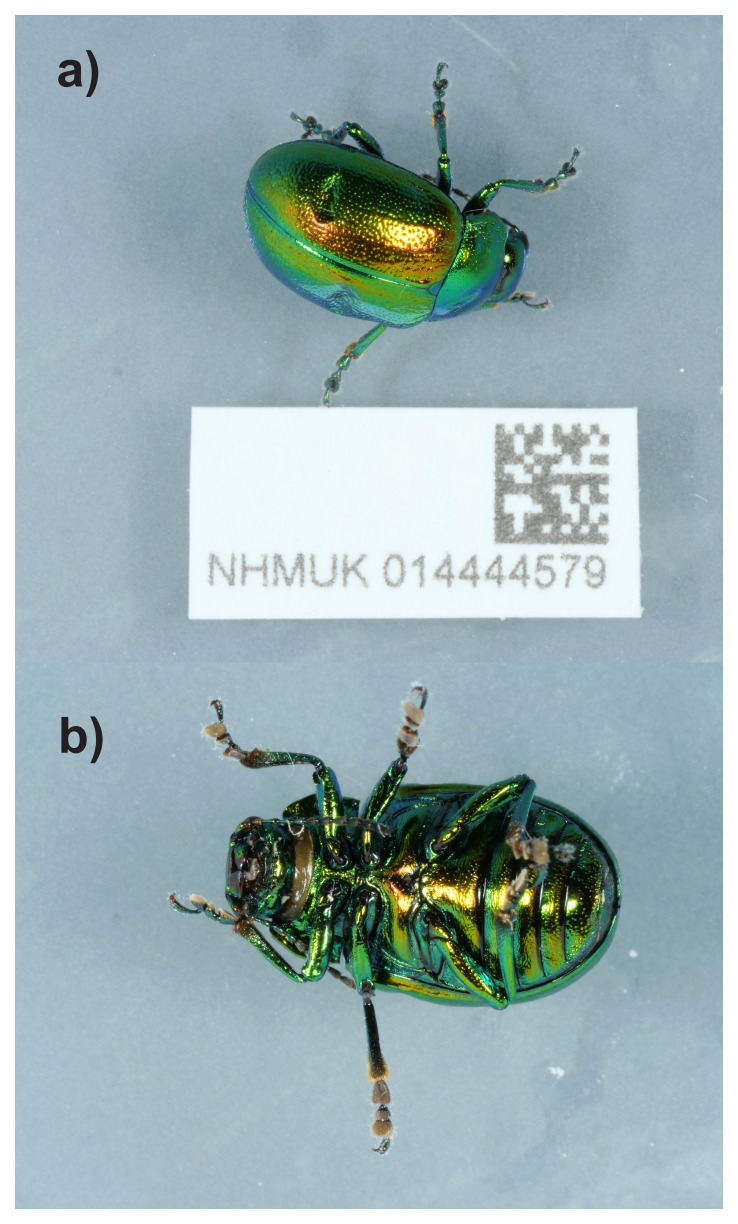
Photographs of the *Chrysolina graminis* (icChrGram1) specimen used for genome sequencing.

The genome of a specimen of *Chrysolina graminis* ( Figure 2) was sequenced using Pacific Biosciences single-molecule HiFi long reads, generating 68.99 Gb (gigabases) from 6.78 million reads, which were used to assemble the genome. GenomeScope analysis estimated the haploid genome size at 1,464.25 Mb, with a heterozygosity of 0.89% and repeat content of 55.28%. These estimates guided expectations for the assembly. Based on the estimated genome size, the sequencing data provided approximately 45 coverage. Hi-C sequencing produced 128.26 Gb from 849.42 million reads, used to scaffold the assembly. RNA sequencing data were also generated and are available in public sequence repositories. Table 1 summarises the specimen and sequencing details.

**Table 1.** Specimen and sequencing data for *Chrysolina graminis*.

Project information			
**Study title**	Chrysolina graminis		
**Umbrella BioProject**	PRJEB73683		
**Species**	*Chrysolina graminis*		
**BioSpecimen**	SAMEA9654295		
**NCBI taxonomy ID**	111401		
Specimen information			
**Technology**	**ToLID**	**BioSample accession**	**Organism part**
**PacBio long read sequencing**	icChrGram1	SAMEA9654372	thorax
**Hi-C sequencing**	icChrGram1	SAMEA9654370	head
**RNA sequencing**	icChrGram3	SAMEA10800184	Whole organism
Sequencing information			
**Platform**	**Run accession**	**Read count**	**Base count (Gb)**
**Hi-C Illumina NovaSeq 6000**	ERR12737291	8.49e+08	128.26
**PacBio Revio**	ERR12736909	4.22e+06	43.51
**PacBio Sequel IIe**	ERR12736910	2.56e+06	25.48
**RNA Illumina NovaSeq 6000**	ERR12737292	7.50e+07	11.32

### Assembly statistics

The genome was assembled into two haplotypes using Hi-C phasing. Haplotype 1 was curated to chromosome level, while haplotype 2 was assembled to scaffold level. The assembly was improved by manual curation, which corrected 193 misjoins or missing joins. These interventions reduced the total assembly length by 1.25%, decreased the scaffold count by 5.38% and increased the scaffold N50 by 1.76%. The final assembly has a total length of 1,467.20 Mb in 650 scaffolds, with 848 gaps, and a scaffold N50 of 131.68 Mb ( Table 2).

**Table 2.** Genome assembly data for *Chrysolina graminis*.

Genome assembly	Haplotype 1	Haplotype 2
Assembly name	icChrGram1.hap1.1	icChrGram1.hap2.1
Assembly accession	GCA_964197785.1	GCA_964197765.1
Assembly level	chromosome	scaffold
Span (Mb)	1,467.20	1,455.42
Number of contigs	1,498	1,150
Number of scaffolds	650	350
Longest scaffold (Mb)	175.9	-
Assembly metrics (benchmark)	Haplotype 1	Haplotype 2
Contig N50 length (≥ 1 Mb)	2.84 Mb	3.0 Mb
Scaffold N50 length (= chromosome N50)	131.68 Mb	129.36 Mb
Consensus quality (QV) (≥ 40)	59.7	60.0
*k*-mer completeness	82.35%	82.42%
Combined *k*-mer completeness (≥ 95%)	98.75%	
BUSCO * (S > 90%; D < 5%)	C:99.0%[S:96.3%,D:2.6%], F:0.3%,M:0.7%,n:2,124	-
Percentage of assembly assigned to chromosomes (≥ 90%)	98.03%	-
Sex chromosomes (localised homologous pairs)	X	-
Organelles (one complete allele)	Mitochondrial genome: 17.07 kb	-

*BUSCO scores based on the endopterygota_odb10 BUSCO set using version 5.5.0. C = complete [S = single copy, D = duplicated], F = fragmented, M = missing, n = number of orthologues in comparison.

**Figure 3.**
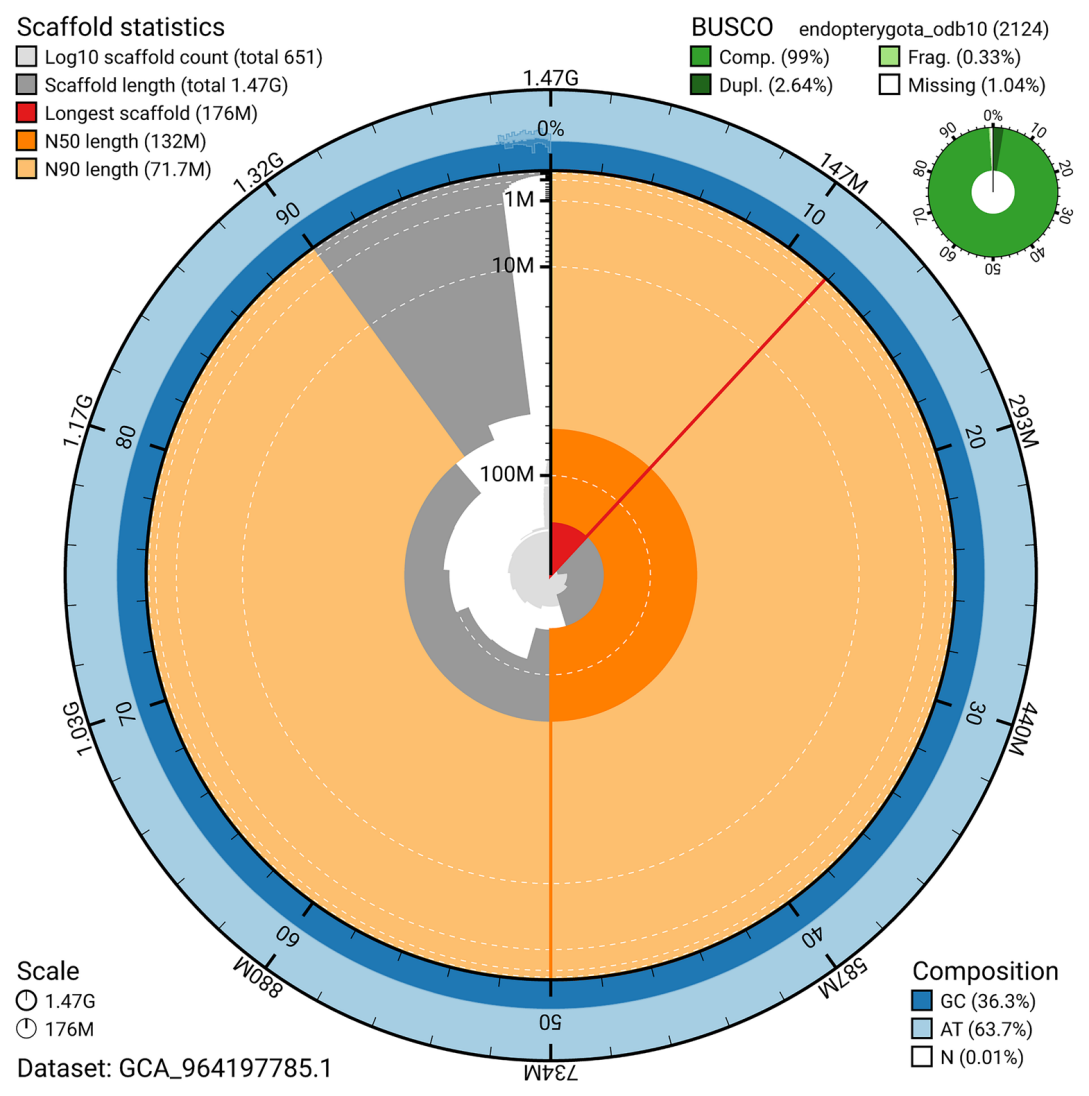
Genome assembly of *Chrysolina graminis*, icChrGram1.hap1.1: metrics. The BlobToolKit snail plot provides an overview of assembly metrics and BUSCO gene completeness. The circumference represents the length of the whole genome sequence, and the main plot is divided into 1,000 bins around the circumference. The outermost blue tracks display the distribution of GC, AT, and N percentages across the bins. Scaffolds are arranged clockwise from longest to shortest and are depicted in dark grey. The longest scaffold is indicated by the red arc, and the deeper orange and pale orange arcs represent the N50 and N90 lengths. A light grey spiral at the centre shows the cumulative scaffold count on a logarithmic scale. A summary of complete, fragmented, duplicated, and missing BUSCO genes in the endopterygota_odb10 set is presented at the top right. An interactive version of this figure is available at https://blobtoolkit.genomehubs.org/view/GCA_964197785.1/dataset/GCA_964197785.1/snail.

**Figure 4.**
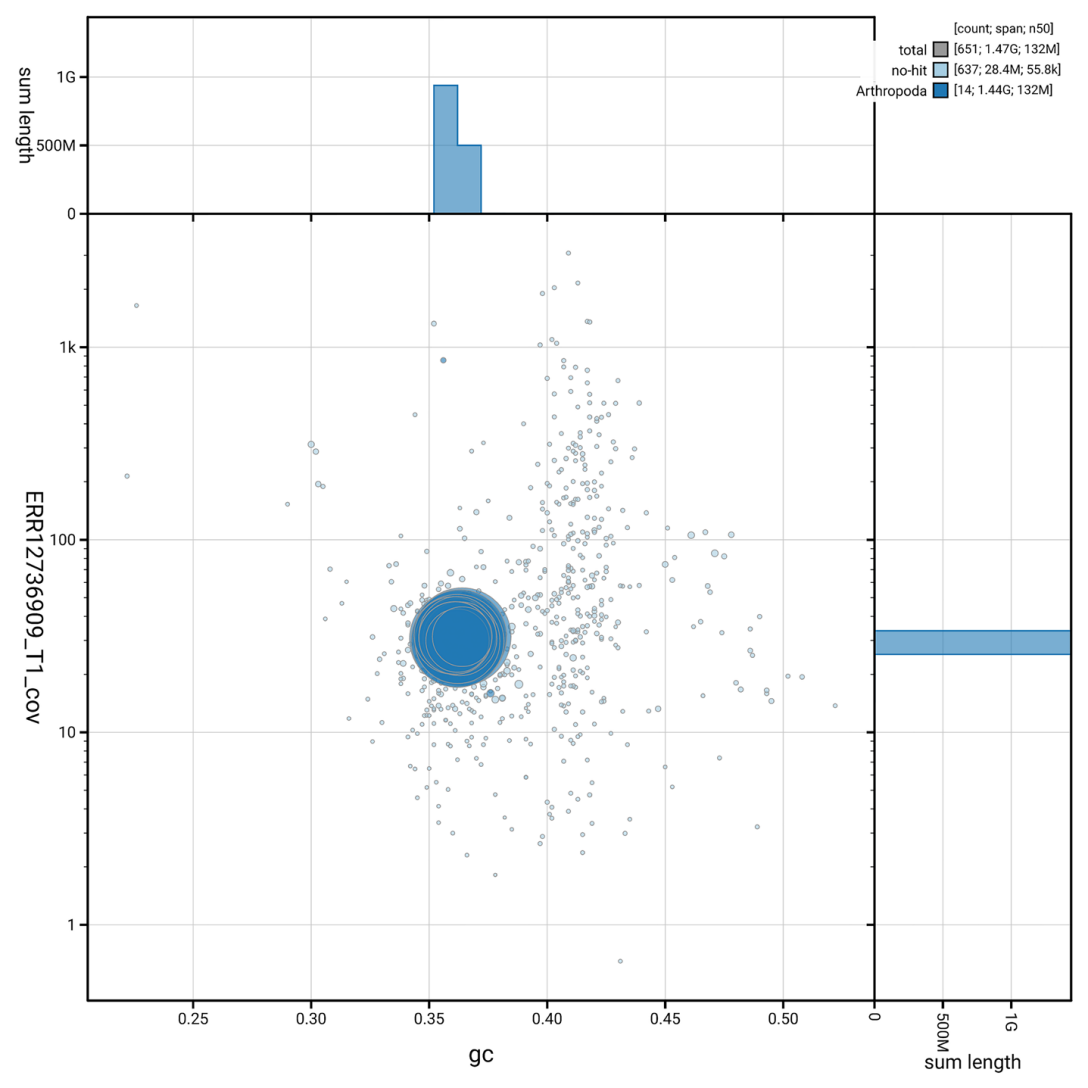
Genome assembly of *Chrysolina graminis*, icChrGram1.hap1.1: BlobToolKit GC-coverage plot. Blob plot showing sequence coverage (vertical axis) and GC content (horizontal axis). The circles represent scaffolds, with the size proportional to scaffold length and the colour representing phylum membership. The histograms along the axes display the total length of sequences distributed across different levels of coverage and GC content. An interactive version of this figure is available at https://blobtoolkit.genomehubs.org/view/GCA_964197785.1/dataset/GCA_964197785.1/blob.

**Figure 5.**
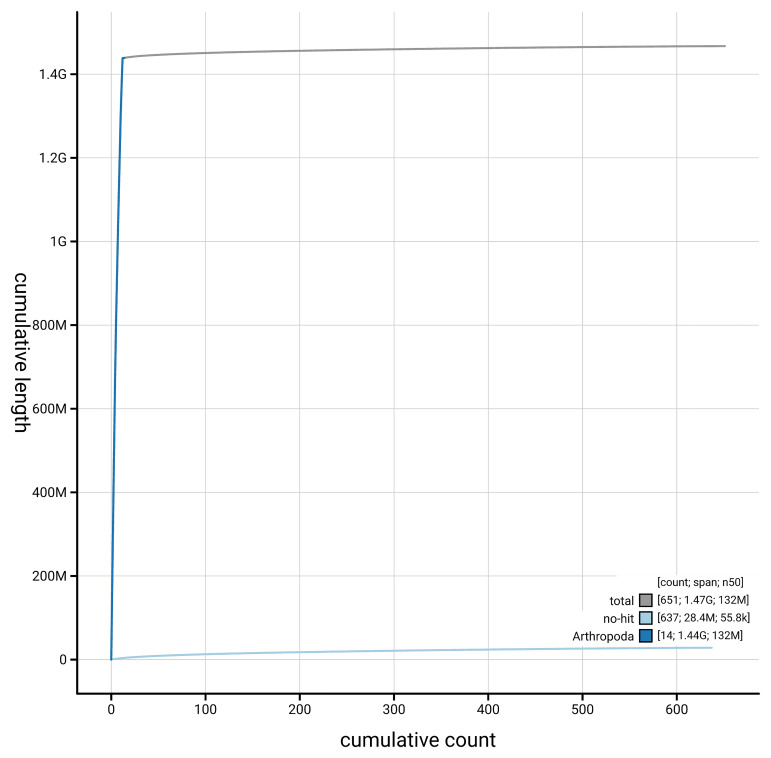
Genome assembly of *Chrysolina graminis,* icChrGram1.hap1.1: BlobToolKit cumulative sequence plot. The grey line shows cumulative length for all scaffolds. Coloured lines show cumulative lengths of scaffolds assigned to each phylum using the buscogenes taxrule. An interactive version of this figure is available at https://blobtoolkit.genomehubs.org/view/GCA_964197785.1/dataset/GCA_964197785.1/cumulative.

The snail plot in Figure 3 provides a summary of the assembly statistics, indicating the distribution of scaffold lengths and other assembly metrics. Figure 4 shows the distribution of scaffolds by GC proportion and coverage. Figure 5 presents a cumulative assembly plot, with separate curves representing different scaffold subsets assigned to various phyla, illustrating the completeness of the assembly.

**Figure 6.**
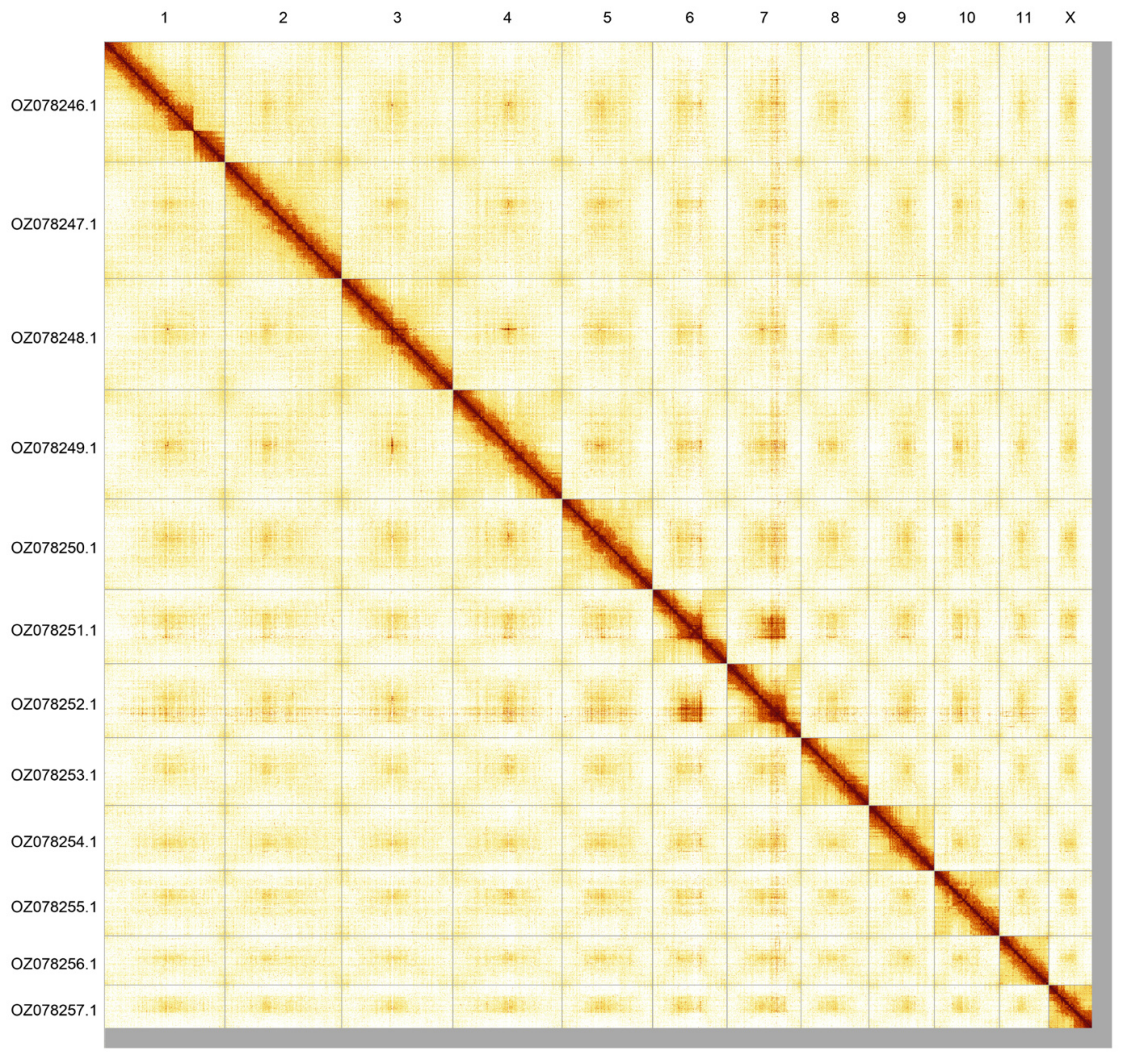
Genome assembly of *Chrysolina graminis*. Hi-C contact map of the icChrGram1.hap1.1 assembly, generated using PretextSnapshot. Chromosomes are shown in order of size and labelled with chromosome numbers (top) and chromosome accession numbers (left).

Most of the assembly sequence (98.03%) was assigned to 12 chromosomal-level scaffolds, representing 11 autosomes and the X sex chromosome. These chromosome-level scaffolds, confirmed by Hi-C data, are named according to size ( Figure 6; Table 3).

**Table 3.** Chromosomal pseudomolecules in the genome assembly of *Chrysolina graminis*, icChrGram1.

INSDC accession	Name	Length (Mb)	GC%
OZ078246.1	1	175.9	36.5
OZ078247.1	2	169.99	36
OZ078248.1	3	162.04	36.5
OZ078249.1	4	158.92	36
OZ078250.1	5	131.68	36
OZ078251.1	6	108.44	36
OZ078252.1	7	107.66	36
OZ078253.1	8	98.83	36.5
OZ078254.1	9	95.08	36
OZ078255.1	10	94.84	36
OZ078256.1	11	71.67	36
OZ078257.1	X	63.26	36.5
OZ078258.1	MT	0.02	23.5

The mitochondrial genome was also assembled. This sequence is included as a contig in the multifasta file of the genome submission and as a standalone record.

### Assembly quality metrics

The estimated Quality Value (QV) and *k*-mer completeness metrics, along with BUSCO completeness scores, were calculated for each haplotype and the combined assembly. The QV reflects the base-level accuracy of the assembly, while *k*-mer completeness indicates the proportion of expected *k*-mers identified in the assembly. BUSCO scores provide a measure of completeness based on benchmarking universal single-copy orthologues.

For haplotype 1, the estimated QV is 59.7, and for haplotype 2, 60.0. When the two haplotypes are combined, the assembly achieves an estimated QV of 59.9. The *k*-mer completeness is 82.35% for haplotype 1 and 82.42% for haplotype 2; and 98.75% for the combined haplotypes. BUSCO v.5.5.0 analysis using the endopterygota_odb10 reference set ( *n* = 2,124) identified 99.0% of the expected gene set (single = 96.3%, duplicated = 2.6%) for haplotype 1.

Table 2 provides assembly metric benchmarks adapted from Rhie *et al.* (2021) and the Earth BioGenome Project Report on Assembly Standards September 2024. The haplotype 1 assembly achieves the EBP reference standard of **6.C.Q59**.

## Methods

### Sample acquisition and DNA barcoding

The specimen used for genome sequencing was an adult female *Chrysolina graminis* (specimen ID NHMUK014444579, ToLID icChrGram1). Another specimen was used for RNA sequencing (specimen ID NHMUK014444578, ToLID icChrGram3). The specimens were collected from Water Fulford Hall, York, England, United Kingdom (latitude 53.928, longitude –1.075), using an aerial net. Both specimens were collected and identified by Geoff Oxford (University of York) and preserved dry freezing (–80 °C).

The initial identification was verified by an additional DNA barcoding process according to the framework developed by Twyford *et al*. (2024). A small sample was dissected from each specimen and stored in ethanol, while the remaining parts were shipped on dry ice to the Wellcome Sanger Institute (WSI) ( Pereira *et al.*, 2022). The tissue was lysed, the COI marker region was amplified by PCR, and amplicons were sequenced and compared to the BOLD database, confirming the species identification ( Crowley *et al.*, 2023). Following whole genome sequence generation, the relevant DNA barcode region was also used alongside the initial barcoding data for sample tracking at the WSI ( Twyford *et al.*, 2024). The standard operating procedures for Darwin Tree of Life barcoding have been deposited on protocols.io ( Beasley *et al.*, 2023).

Metadata collection for samples adhered to the Darwin Tree of Life project standards described by Lawniczak *et al*. (2022).

### Nucleic acid extraction

The workflow for high molecular weight (HMW) DNA extraction at the Wellcome Sanger Institute (WSI) Tree of Life Core Laboratory includes a sequence of procedures: sample preparation and homogenisation, DNA extraction, fragmentation and purification ( Howard *et al.*, 2025). Detailed protocols are available on protocols.io ( Denton *et al.*, 2023b). The icChrGram1 sample was prepared for DNA extraction by weighing and dissecting it on dry ice ( Jay *et al.*, 2023).

Tissue from the thorax was homogenised using a PowerMasher II tissue disruptor ( Denton *et al.*, 2023a). HMW DNA was extracted in the WSI Scientific Operations core using the Automated MagAttract v2 protocol ( Oatley *et al.*, 2023a). For ultra-low input (ULI) PacBio sequencing, DNA was fragmented using the Covaris g-TUBE method ( Oatley *et al.*, 2023b). Sheared DNA was purified by solid-phase reversible immobilisation, using AMPure PB beads to eliminate shorter fragments and concentrate the DNA ( Strickland *et al.*, 2023). The concentration of the sheared and purified DNA was assessed using a Nanodrop spectrophotometer and Qubit Fluorometer using the Qubit dsDNA High Sensitivity Assay kit. Fragment size distribution was evaluated by running the sample on the FemtoPulse system.

RNA was extracted from tissue of icChrGram3 in the Tree of Life Laboratory at the WSI using the RNA Extraction: Automated MagMax™ *mir*Vana protocol ( do Amaral *et al.*, 2023). The RNA concentration was assessed using a Nanodrop spectrophotometer and a Qubit Fluorometer using the Qubit RNA Broad-Range Assay kit. Analysis of the integrity of the RNA was done using the Agilent RNA 6000 Pico Kit and Eukaryotic Total RNA assay.

### Hi-C sample preparation and crosslinking

Hi-C data were generated from the head of the icChrGram1 sample using the Arima-HiC v2 kit (Arima Genomics) with 20–50 mg of frozen tissue (stored at –80 °C). As per manufacturer’s instructions, tissue was fixed, and the DNA crosslinked using a TC buffer with a final formaldehyde concentration of 2%. The tissue was then homogenised using the Diagnocine Power Masher-II. The crosslinked DNA was digested using a restriction enzyme master mix, then biotinylated and ligated. A clean up was performed with SPRIselect beads prior to library preparation. DNA concentration was quantified using the Qubit Fluorometer v4.0 (Thermo Fisher Scientific) and Qubit HS Assay Kit, and sample biotinylation percentage was estimated using the Arima-HiC v2 QC beads.

### Library preparation and sequencing

Library preparation and sequencing were performed at the WSI Scientific Operations core.

***PacBio HiFi (ULI)***

Ultra-low input libraries were prepared using PacBio SMRTbell® Express Template Prep Kit 2.0 and PacBio SMRTbell® gDNA Sample Amplification Kit. To begin, samples were normalised to 20 ng of DNA. Initial removal of single-strand overhangs, DNA damage repair, and end repair/A-tailing were performed per manufacturer’s instructions. From the SMRTbell® gDNA Sample Amplification Kit, amplification adapters were then ligated. A 0.85X pre-PCR clean-up was performed with Promega ProNex beads and the sample was then divided into two for a dual PCR. PCR reactions A and B each followed the PCR programs as described in the manufacturer’s protocol. A 0.85X post-PCR clean-up was performed with ProNex beads for PCR reactions A and B and DNA concentration was quantified using the Qubit Fluorometer v4.0 (Thermo Fisher Scientific) and Qubit HS Assay Kit and fragment size analysis was carried out using the Agilent Femto Pulse Automated Pulsed Field CE Instrument (Agilent Technologies) and gDNA 55kb BAC analysis kit. PCR reactions A and B were then pooled, ensuring the total mass was ≥500 ng in 47.4 μl. The pooled sample then repeated the process for DNA damage repair, end repair/A-tailing and additional hairpin adapter ligation. A 1X clean-up was performed with ProNex beads and DNA concentration was quantified using the Qubit and fragment size analysis was carried out using the Agilent Femto Pulse Automated Pulsed Field CE Instrument (Agilent Technologies). Size selection was performed using Sage Sciences' PippinHT system with target fragment size determined by analysis from the Femto Pulse, usually a value between 4000 and 9000 bp. Size selected libraries were then cleaned-up using 1.0X ProNex beads and normalised to 2 nM before proceeding to sequencing.

Samples were sequenced using the Sequel IIe system as well as on a Revio instrument (Pacific Biosciences, California, USA). The concentration of the library loaded onto the Sequel IIe was in the range 40–135 pM. On the Revio, prepared libraries were normalised to 2 nM, and 15 μL was used for making complexes. Primers were annealed and polymerases were bound to create circularised complexes according to manufacturer’s instructions. The complexes were purified with the 1.2X clean up with SMRTbell beads. The purified complexes were then diluted to the Revio loading concentration (in the range 200–300 pM), and spiked with a Revio sequencing internal control. Samples were sequenced on Revio 25M SMRT cells (Pacific Biosciences, California, USA). The SMRT link software, a PacBio web-based end-to-end workflow manager, was used to set-up and monitor the run, as well as perform primary and secondary analysis of the data upon completion.

***Hi-C***

For Hi-C library preparation, the biotinylated DNA constructs were fragmented using a Covaris E220 sonicator and size-selected to 400–600 bp using SPRISelect beads. DNA was then enriched using Arima-HiC v2 Enrichment beads. The NEBNext Ultra II DNA Library Prep Kit (New England Biolabs) was used for end repair, A-tailing, and adapter ligation, following a modified protocol in which library preparation is carried out while the DNA remains bound to the enrichment beads. PCR amplification was performed using KAPA HiFi HotStart mix and custom dual-indexed adapters (Integrated DNA Technologies) in a 96-well plate format. Depending on sample concentration and biotinylation percentage determined at the crosslinking stage, samples were amplified for 10–16 PCR cycles. Post-PCR clean-up was carried out using SPRISelect beads. The libraries were quantified using the Accuclear Ultra High Sensitivity dsDNA Standards Assay kit (Biotium) and normalised to 10 ng/μL before sequencing. Hi-C sequencing was performed on the Illumina NovaSeq 6000.

***RNA***

Poly(A) RNA-Seq libraries were prepared using the NEBNext ^®^ Ultra™ II Directional RNA Library Prep Kit for Illumina (New England Biolabs), following the manufacturer’s instructions. Poly(A) mRNA in the total RNA solution was isolated using oligo(dT) beads, converted to cDNA, and uniquely indexed; 14 PCR cycles were performed. Libraries were size-selected to produce fragments between 100–300 bp. Libraries were quantified, normalised, pooled to a final concentration of 2.8 nM, and diluted to 150 pM for loading. Sequencing was carried out on the Illumina NovaSeq 6000.

### Genome assembly, curation and evaluation

***Assembly***

Prior to assembly of the PacBio HiFi reads, a database of *k*-mer counts ( *k* = 31) was generated from the filtered reads using FastK. GenomeScope2 ( Ranallo-Benavidez *et al.*, 2020) was used to analyse the *k*-mer frequency distributions, providing estimates of genome size, heterozygosity, and repeat content.

The HiFi reads were assembled using Hifiasm in Hi-C phasing mode ( Cheng *et al.*, 2021; Cheng *et al.*, 2022), resulting in a pair of haplotype-resolved assemblies. The Hi-C reads ( Rao *et al.*, 2014) were mapped to the primary contigs using bwa-mem2 ( Vasimuddin *et al.*, 2019). The contigs were further scaffolded with Hi-C data in YaHS ( Zhou *et al.*, 2023), using the --break option for handling potential misassemblies. The scaffolded assemblies were evaluated using Gfastats ( Formenti *et al.*, 2022), BUSCO ( Manni *et al.*, 2021) and MERQURY.FK ( Rhie *et al.*, 2020).

The mitochondrial genome was assembled using MitoHiFi ( Uliano-Silva *et al.*, 2023), which runs MitoFinder ( Allio *et al.*, 2020) and uses these annotations to select the final mitochondrial contig and to ensure the general quality of the sequence.

***Assembly curation***

The assembly was decontaminated using the Assembly Screen for Cobionts and Contaminants (ASCC) pipeline. Flat files and maps used in curation were generated via the TreeVal pipeline ( Pointon *et al.*, 2023). Manual curation was conducted primarily in PretextView ( Harry, 2022) and HiGlass ( Kerpedjiev *et al.*, 2018), with additional insights provided by JBrowse2 ( Diesh *et al.*, 2023). Scaffolds were visually inspected and corrected as described by Howe *et al*. (2021). Any identified contamination, missed joins, and mis-joins were amended, and duplicate sequences were tagged and removed. The curation process is documented at https://gitlab.com/wtsi-grit/rapid-curation. PretextSnapshot was used to generate a Hi-C contact map of the final assembly.

***Assembly quality assessment***

The Merqury.FK tool ( Rhie *et al.*, 2020), run in a Singularity container ( Kurtzer *et al.*, 2017), was used to evaluate *k*-mer completeness and assembly quality for both haplotypes using the *k*-mer databases ( *k* = 31) computed prior to genome assembly. The analysis outputs included assembly QV scores and completeness statistics.

The genome was analysed using the BlobToolKit pipeline, a Nextflow ( Di Tommaso *et al.*, 2017) implementation of the earlier Snakemake BlobToolKit pipeline ( Challis *et al.*, 2020). The pipeline aligns PacBio reads using minimap2 ( Li, 2018) and SAMtools ( Danecek *et al.*, 2021) to generate coverage tracks. Simultaneously, it queries the GoaT database ( Challis *et al.*, 2023) to identify relevant BUSCO lineages and runs BUSCO ( Manni *et al.*, 2021). For the three domain-level BUSCO lineages, BUSCO genes are aligned to the UniProt Reference Proteomes database ( Bateman *et al.*, 2023) using DIAMOND blastp ( Buchfink *et al.*, 2021). The genome is divided into chunks based on the density of BUSCO genes from the closest taxonomic lineage, and each chunk is aligned to the UniProt Reference Proteomes database with DIAMOND blastx. Sequences without hits are chunked using seqtk and aligned to the NT database with blastn ( Altschul *et al.*, 1990). The BlobToolKit suite consolidates all outputs into a blobdir for visualisation. The BlobToolKit pipeline was developed using nf-core tooling ( Ewels *et al.*, 2020) and MultiQC ( Ewels *et al.*, 2016), with package management via Conda and Bioconda ( Grüning *et al.*, 2018), and containerisation through Docker ( Merkel, 2014) and Singularity ( Kurtzer *et al.*, 2017).

Table 4 contains a list of relevant software tool versions and sources.

**Table 4.** Software tools: versions and sources.

Software tool	Version	Source
BLAST	2.14.0	ftp://ftp.ncbi.nlm.nih.gov/blast/executables/blast+/
BlobToolKit	4.3.9	https://github.com/blobtoolkit/blobtoolkit
BUSCO	5.5.0	https://gitlab.com/ezlab/busco
bwa-mem2	2.2.1	https://github.com/bwa-mem2/bwa-mem2
DIAMOND	2.1.8	https://github.com/bbuchfink/diamond
fasta_windows	0.2.4	https://github.com/tolkit/fasta_windows
FastK	666652151335353eef2fcd58880bcef5bc2928e1	https://github.com/thegenemyers/FASTK
GenomeScope2.0	2.0.1	https://github.com/tbenavi1/genomescope2.0
Gfastats	1.3.6	https://github.com/vgl-hub/gfastats
GoaT CLI	0.2.5	https://github.com/genomehubs/goat-cli
Hifiasm	0.19.8-r603	https://github.com/chhylp123/hifiasm
HiGlass	44086069ee7d4d3f6f3f0012569789ec138f42b84aa44357826c0b6753eb28de	https://github.com/higlass/higlass
MerquryFK	d00d98157618f4e8d1a9190026b19b471055b22e	https://github.com/thegenemyers/MERQURY.FK
Minimap2	2.24-r1122	https://github.com/lh3/minimap2
MitoHiFi	3	https://github.com/marcelauliano/MitoHiFi
MultiQC	1.14, 1.17, and 1.18	https://github.com/MultiQC/MultiQC
Nextflow	23.10.0	https://github.com/nextflow-io/nextflow
PretextSnapshot	-	https://github.com/sanger-tol/PretextSnapshot
PretextView	0.2.5	https://github.com/sanger-tol/PretextView
samtools	1.19.2	https://github.com/samtools/samtools
sanger-tol/ascc	0.1.0	https://github.com/sanger-tol/ascc
sanger-tol/blobtoolkit	0.6.0	https://github.com/sanger-tol/blobtoolkit
Seqtk	1.3	https://github.com/lh3/seqtk
Singularity	3.9.0	https://github.com/sylabs/singularity
TreeVal	1.2.0	https://github.com/sanger-tol/treeval
YaHS	1.2a.2	https://github.com/c-zhou/yahs

### Wellcome Sanger Institute – Legal and Governance

The materials that have contributed to this genome note have been supplied by a Darwin Tree of Life Partner. The submission of materials by a Darwin Tree of Life Partner is subject to the **‘Darwin Tree of Life Project Sampling Code of Practice’**, which can be found in full on the Darwin Tree of Life website here. By agreeing with and signing up to the Sampling Code of Practice, the Darwin Tree of Life Partner agrees they will meet the legal and ethical requirements and standards set out within this document in respect of all samples acquired for, and supplied to, the Darwin Tree of Life Project.

Further, the Wellcome Sanger Institute employs a process whereby due diligence is carried out proportionate to the nature of the materials themselves, and the circumstances under which they have been/are to be collected and provided for use. The purpose of this is to address and mitigate any potential legal and/or ethical implications of receipt and use of the materials as part of the research project, and to ensure that in doing so we align with best practice wherever possible. The overarching areas of consideration are:

•      Ethical review of provenance and sourcing of the material

•      Legality of collection, transfer and use (national and international)

Each transfer of samples is further undertaken according to a Research Collaboration Agreement or Material Transfer Agreement entered into by the Darwin Tree of Life Partner, Genome Research Limited (operating as the Wellcome Sanger Institute), and in some circumstances other Darwin Tree of Life collaborators.

## Data Availability

European Nucleotide Archive: Chrysolina graminis. Accession number PRJEB73683; https://identifiers.org/ena.embl/PRJEB73683. The genome sequence is released openly for reuse. The *Chrysolina graminis* genome sequencing initiative is part of the Darwin Tree of Life Project (PRJEB40665) and the Sanger Institute Tree of Life Programme (PRJEB43745). All raw sequence data and the assembly have been deposited in INSDC databases. The genome will be annotated using available RNA-Seq data and presented through the Ensembl pipeline at the European Bioinformatics Institute. Raw data and assembly accession identifiers are reported in Table 1 and Table 2.

## Author information

Members of the Natural History Museum Genome Acquisition Lab are listed here: https://doi.org/10.5281/zenodo.12159242.

Members of the Darwin Tree of Life Barcoding collective are listed here: https://doi.org/10.5281/zenodo.12158331.

Members of the Wellcome Sanger Institute Tree of Life Management, Samples and Laboratory team are listed here: https://doi.org/10.5281/zenodo.12162482.

Members of Wellcome Sanger Institute Scientific Operations: Sequencing Operations are listed here: https://doi.org/10.5281/zenodo.12165051.

Members of the Wellcome Sanger Institute Tree of Life Core Informatics team are listed here: https://doi.org/10.5281/zenodo.12160324.

Members of the Tree of Life Core Informatics collective are listed here: https://doi.org/10.5281/zenodo.12205391.

Members of the Darwin Tree of Life Consortium are listed here: https://doi.org/10.5281/zenodo.4783558.
