# Supplementary material for: The genome sequence of the Tansy Beetle, Chrysolina graminis (Linnaeus, 1758)
Source: Wellcome Open Res. 2025 Jun 2;10:296. [Version 1] doi: 10.12688/wellcomeopenres.24287.1 (PMC12669981; doi:10.12688/wellcomeopenres.24287.1)
Supplement: Peer review history

# Reviewer report: Reviewer response for version 1

**DOI:** 10.21956/wellcomeopenres.26785.r139480

**Published:** 2025-12-01

**Authors:** Laurence Despres

**Affiliations:** 1 Universite Savoie Mont Blanc, Auvergne-Rhône-Alpes, France; 2 Université Grenoble Alpes, Laboratoire d'Ecologie Alpine (Ringgold ID: 56837), Grenoble, Auvergne-Rhône-Alpes, France

The authors report on the chromosome-level genome assembly of a female specimen of *Chrysolina graminis* (Tansy Beetle; Chrysomelidae), an iridescent green or bronze leaf-beetle widely distributed throughout the Palaearctic. The assembly contains two haplotypes with most of haplotype 1 scaffolded into 12 chromosomes, including the X sex chromosome. Haplotype 2 was assembled to scaffold level. The mitochondrial genome has also been assembled.

 The quality of the assembly was further assessed by the proportion of complete BUSCO genes recovered, which is very high (99.0% of the expected gene set of the endopterygota_odb10 reference database). RNA sequencing data were also generated. The genome assembly is of good quality and was constructed using appropriate methods: Pac Bio HiFi long reads (45 coverage) and Hi-C data to confirm scaffolding, following the high standard of the Darwin Tree of Life Project for results presentation.

Are sufficient details of methods and materials provided to allow replication by others?

Yes

Is the rationale for creating the dataset(s) clearly described?

Yes

Are the datasets clearly presented in a useable and accessible format?

Yes

Are the protocols appropriate and is the work technically sound?

Yes

Reviewer Expertise:

evolutionary ecology

I confirm that I have read this submission and believe that I have an appropriate level of expertise to confirm that it is of an acceptable scientific standard.

# Reviewer report: Reviewer response for version 1

**DOI:** 10.21956/wellcomeopenres.26785.r125715

**Published:** 2025-07-14

**Authors:** Komal Kumar Bollepogu Raja Raja

**Affiliations:** 1 Baylor College of Medicine, Houston, Texas, USA

The article by Oxford G.S. presents the genome sequence of the species *Chrysolina* *graminis*. The assembly has 2 haplotypes scaffolded into 12 chromosomal molecules. The ecological and morphological aspects of this species is well presented in the background. The contig assembly metrics look good along with impressive k-mer completeness and BUSCO scores suggesting that the genome is high quality. The Hi-C contact map shows clear diagonal signal with minimal noise. There are a few off-diagonal interactions. The experimental methods are robust and allow for reproducibility. Overall, the genome sequence is high quality and is a great genomic resource.

 Minor comments

 1. Under the subheading Sequencing data, should it be 45X coverage instead of 45 coverage? Please correct this error.

Are sufficient details of methods and materials provided to allow replication by others?

Yes

Is the rationale for creating the dataset(s) clearly described?

Yes

Are the datasets clearly presented in a useable and accessible format?

Yes

Are the protocols appropriate and is the work technically sound?

Yes

Reviewer Expertise:

Bioinformatics, genomics, single cell genomics and Neurobiology

I confirm that I have read this submission and believe that I have an appropriate level of expertise to confirm that it is of an acceptable scientific standard.
